# Exploring the ancient roots and modern global brews of tea and herbal beverages: A comprehensive review of origins, types, health benefits, market dynamics, and future trends

**DOI:** 10.1002/fsn3.4346

**Published:** 2024-07-21

**Authors:** Hisham‐Sultan‐Alkatib Huda, Nazia Binti Abdul Majid, Yeng Chen, Mohd Adnan, Syed Amir Ashraf, Marek Roszko, Marcin Bryła, Marek Kieliszek, Sreenivasan Sasidharan

**Affiliations:** ^1^ Institute for Research in Molecular Medicine University Sains Malaysia Penang Malaysia; ^2^ Institute of Biological Sciences. Faculty of Science University of Malaya Kuala Lumpur Malaysia; ^3^ Department of Oral & Craniofacial Sciences, Faculty of Dentistry University of Malaya Kuala Lumpur Malaysia; ^4^ Department of Biology, College of Science University of Ha'il Ha'il Saudi Arabia; ^5^ Department of Clinical Nutrition, College of Applied Medical Sciences University of Ha'il Ha'il Saudi Arabia; ^6^ Department of Food Safety and Chemical Analysis Prof. Wacław Dąbrowski Institute of Agricultural and Food Biotechnology—State Research Institute Warsaw Poland; ^7^ Department of Food Biotechnology and Microbiology, Institute of Food Sciences Warsaw University of Life Sciences—SCGW Warsaw Poland

**Keywords:** beverages, blended tea, *Camellia sinensis*, flavonoids, herbal tea, polyphenol

## Abstract

Tea, a culturally significant beverage, originated around 2700 B.C. in ancient Chinese civilization, with a profound understanding of its therapeutic properties. Herbal medicines from diverse plant sources have been esteemed for their phytochemical content. Today, tea's appeal spans the globe, with various processing techniques creating distinct tea varieties. This review article comprehensively explores tea and herbal teas, encompassing their origins, types, trade history, health benefits, chemical composition, and market and future dynamics. This review examines tea's evolution from ancient China to its global significance and analyzes the impact of tea trade routes on cultural exchanges and trade dynamics. The review covers conventional teas (black, green, and oolong), blended teas, and herbal teas. It primarily focuses on herbal beverages' chemical composition and active components derived from diverse plants and botanicals, highlighting their traditional uses and health‐promoting applications. The review provides valuable insights into the dynamic herbal tea market, growth, consumer preferences, industry trends, and future aspects of the herbal beverage. Additionally, it explores the proper classification and preparation of herbal drinks for maximum benefits, shedding light on tea manufacturing and preparation processes. This review is a valuable resource for tea enthusiasts, health‐conscious individuals, and industry stakeholders, offering profound insights into teas and their multifaceted allure.

## Graphical abstract

Explores herbal teas and herbal beverages for their origins, types and trade history. Valuable insights into the market dynamic and future prospects of herbal beverage. Herbal beverages' bioactive components and their health‐promoting applications

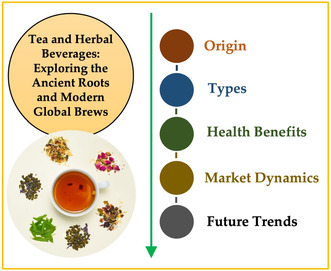

## 1 INTRODUCTION

Tea, a time‐honored beverage renowned for its cultural significance, has a history deeply rooted in human civilization. Defined as an infusion derived from the young leaves, leaf buds, and internodes of *Camellia sinensis* or *Camellia assamica* (Hicks, 2001), tea's origins can be traced back to approximately 2700 B.C., with its early development primarily centered in Chinese culture (Pan et al., 2022). Throughout history, natural items, particularly plants and their diverse constituents, have been integral to the practice of treating illnesses and maintaining human well‐being. Herbal medicines, sourced from leaves, flowers, fruits, barks, roots, and rhizomes, have been esteemed for their abundant phytochemical content, offering a broad spectrum of culinary and medicinal applications (Tipduangta et al., 2019). The early Chinese civilizations, dating as far back as 400–5000 years ago, demonstrated a profound understanding of tea's potential therapeutic properties, acknowledging its capacity to address and prevent various human ailments (Erling Hoh, 2009). Presently, tea has transcended geographical boundaries, finding favor among millions of consumers worldwide, and perpetuating its timeless allure across generations. Over time, tea's allure has driven the diversification of tea varieties, each characterized by distinct processing techniques. Green, black, and oolong teas constitute the primary tea types, distinguished by varying levels of fermentation that influence their flavor profiles. Recent years have witnessed a notable surge in interest surrounding herbal teas, also known as tisanes. Derived from an extensive array of plants and botanicals, herbal teas have garnered acclaim for their health benefits and traditional medicinal attributes. An abundant corpus of research literature furnishes ample reports substantiating the potential of tea consumption in mitigating the risk of cardiovascular and debilitating diseases, propelling the growth of the tea‐based products market with a steadfast commitment to food safety and health standards (Khan & Mukhtar, 2013; Reygaert, 2017). This review article endeavors to thoroughly explore the historical origins of tea, delving into its trade history, and providing comprehensive insight into the various common tea types. A particular emphasis will be placed on the burgeoning popularity and health benefits of herbal teas. The herbal tea market will be examined closely, including the classification and preparation methods, alongside broader considerations encompassing tea manufacturing and preparation. Throughout this investigation, the potential health benefits of herbal teas will be accentuated, drawing from empirical evidence reported in various medicinal research studies. Our examination of academic research literature seeks to reinforce the understanding of herbal teas' therapeutic potential, to inspire further exploration into the merits of integrating these venerable beverages into a health‐conscious lifestyle.

## 2 METHODS

For the literature search, research and review articles published between 1980 and 2024 were examined, and sourced from Scopus, PubMed, Web of Science, and Google Scholar. The search strategy incorporated keywords such as “Tea and herbal beverages,” “Origins of tea and herbal beverages,” “Types of tea and herbal beverages,” “Health benefits of tea and herbal beverages,” “Market dynamics of tea and herbal beverages,” and “Future trends of tea and herbal beverages,” ensuring a comprehensive review of studies analyzing the origins, types, health benefits, market dynamics, and future trends of tea and herbal beverages. Each study was assessed for inclusion based on its relevance to tea and herbal beverages, excluding duplicates, meta‐analyses, insufficiently detailed studies, and those not in English. Additionally, original articles from January 1980 to April 2024 were included, focusing on the origins, types, health benefits, market dynamics, and future trends of tea and herbal beverages.

## 3 TEA HISTORY AND ORIGIN

According to ancient legend, it is believed that Shen Nung, the second emperor of China, accidentally discovered tea around 2737 BCE (Pan et al., 2022). One day, he was unwinding in the yard with a cup of boiling water after a substantial supper. A few leaves from a neighboring tree fell into the cup at that same moment. He took a sip without realizing it. The habit of drinking tea was established as a result of his enjoyment of its flavor and perception that it significantly reduced the amount of discomfort he was experiencing at the time. According to an Indian myth, Buddha began to feel sleepy in the fifth year of a 7‐year sleepless meditation. He quickly plucked a few leaves from a nearby shrub and began chewing them. Miraculously, his fatigue dissipated. The plant happened to be an untamed tea tree (Selvakumar & Jeyaselvam, 2012).

Father Jasper de Cruz, a Portuguese Jesuit missionary, was the first European to experience tea and write about it in 1560. The Dutch brought several tea and tea customs to New Amsterdam city around 1650 (which eventually transformed into the city of New York). In 1657, at Garway's Coffee House in London, England, the first tea was introduced and consumed as a beverage with health‐promoting properties. The first retail tea was offered by John Horniman in lead‐lined, sealed containers in 1826. Twinings of England, a company that originated in England and was founded in 1706, has a rich history of tea blending and trading. It is recognized as one of the world's oldest and most esteemed tea companies. In 1870, Twinings of England began blending tea to achieve consistency in their tea products. Richard Blechynden, an Englishman, invented iced tea in 1904 amid a period of scorching temperatures at the St. Louis World Fair. Tea bags were accidentally invented by tea importer Thomas Sullivan of New York in 1908 when he supplied tea to customers in small silk bags that were inadvertently steeped whole. In 1953, the first instant tea in the world was unveiled. Today, green tea is drunk in Asia, while black tea is consumed in North Africa, North America, and Europe (Stander et al., 2019).

## 4 TEA TRADE HISTORY

After being consumed in China for thousands of years as a medicinal beverage made by steeping fresh leaves in boiled water, tea became a common beverage and the cultivation and processing of tea started about the third century C.E. (Wambulwa et al., 2021; Yang et al., 2014). In 350 C.E., the earliest description of how to plant, prepare, and drink was published. Tea cultivation has a long history, with the first tea seeds being introduced to Japan around 800 A.D., and agriculture based on tea establishing itself by the 13th century. Tea cultivation was introduced to the Formosa (Taiwan) Island in 1810 by Chinese immigrants from Amoy. The Dutch brought Japanese seeds and workers to Java in 1826 and introduced Chinese seeds, laborers, and equipment to the highlands near the border between the Indian state of Assam and Burma in 1833. Tea trees were discovered in this region in 1824. In 1836 and 1867, respectively, the cultivation of tea in India and Ceylon (Sri Lanka) was introduced by the British, initially using Chinese seeds but eventually switching to Assam plant seeds (Sivasubramaniam, 2022).

In 1610, the Dutch East India Company successfully delivered the initial consignment of Chinese tea to Europe, while in 1669, the English East India Company transported Chinese tea to the London market. Over time, tea produced on British estates in Ceylon and India also became popular. By the late 19th and early 20th centuries, tea was being grown in non‐Asian nations, such as Russian Georgia, Sumatra, and Iran, as well as several African countries including Malawi, Natal, Congo, Uganda, Kenya, Mozambique, and Tanzania. South American countries like Argentina, Brazil, and Peru, as well as Queensland in Australia, also began growing tea (Sivasubramaniam, 2022).

A wide variety of teas are produced throughout the Asian region. As a result, Asia now holds a significant share in every global tea importing market, owing to its reputation for producing high‐quality teas that are well regarded in global markets (Hicks, 2009). Tea is also extensively produced in the Near East, South America, and Africa, contributing to significant tea production worldwide. It is consumed regularly throughout the day by large populations in various regions including Asia, the United Kingdom, the European Union, the Middle East, Africa, and the Commonwealth of Independent States (CIS) (Hicks, 2001).

## 5 COMMON TYPES OF TEA

There are several significant conventional teas, including black tea, green tea, yellow tea, red tea, dark tea, and white tea. Among these varieties, green, black, and oolong teas stand out as the most widely produced and consumed in different regions around the world. Despite being derived from the same plant (*C. sinensis*), these three types differ due to the distinct degrees of fermentation they undergo during processing (Zhang et al., 2019). Black tea dominates global tea production, accounting for 78%, while green tea and oolong tea hold 20% and 2% of the total production, respectively (Hayat et al., 2015).

### 5.1 Black tea

Black tea, the most widely traded type, undergoes full fermentation during the manufacturing process, leading to significant biochemical reactions in the tea leaves, including distortion and fermentation stages. These reactions result in the formation of oxidized and polymerized components, which contribute to the distinct flavor and aroma of black tea (Yamanishi, 1995). Notable examples of black teas include Pekoe and Orange Pekoe, which are prepared from the top leaves of the tea plant. Orange Pekoe is sourced from Sri Lanka or India (Perera et al., 2020). The production of black tea involves two main methods: conventional processing and cut, tear, and curl (CTC) processing. Conventional processing, originally done by hand rolling but now mechanized, and CTC processing, which utilizes machines to tear the leaves, both accelerate the fermentation process (Aaqil et al., 2023). Black tea holds a dominant position in the global tea market (Yong‐mei et al., 2022). It is favored for its mild flavor, bright red color, unique taste, and rich nutritional content (Gao et al., 2022).

### 5.2 Green tea

Green tea is unfermented and retains its original polyphenols due to minimal processing. The leaves are withered and steamed to preserve their natural chemical composition. Green tea is particularly popular in the Far East and some Middle Eastern countries (Yamanishi, 1995).

### 5.3 Oolong tea

Oolong tea is partially fermented, offering a flavor profile between green and black tea. The leaves are withered, crushed, and allowed to undergo partial oxidation before being heated and dried (Ho et al., 2018). Oolong tea is also widely consumed in the Far East.

Each type of tea has its own distinctive taste, aroma, and health benefits due to variations in processing and chemical composition (Ying Gao et al., 2020). Consumers worldwide enjoy these teas for their diverse flavor profiles and potential health‐promoting properties. Tea remains a beverage cherished worldwide, and its brewing techniques vary across different cultures. Brewing times, infusion strengths, and the use of teabags can differ among countries, reflecting the diverse preferences of tea drinkers (Czarniecka‐Skubina et al., 2022).

In addition to the traditional types of tea, tea blending has become a popular practice in the tea industry. Blending tea involves the skillful mixing of different varieties or grades of tea from various states or regions to create unique and distinctive blends. This art form allows tea producers to cater to diverse consumer preferences by achieving specific flavor profiles, colors, and aromas. Tea blends are often crafted to achieve a balanced and harmonious taste, taking into account factors such as the origin of the teas, their processing methods, and the desired characteristics of the final product. Blending allows producers to create signature blends that stand out in the market and meet the preferences of their target consumers. Some common examples of tea blends include:

### 5.4 Earl gray

A classic and widely recognized tea blend, Earl Gray combines black tea with the essence of bergamot, a citrus fruit, giving it a distinct and fragrant flavor.

### 5.5 English breakfast

Another popular blend, English breakfast typically includes a mix of Assam, Ceylon, and Kenyan black teas, known for their robust and malty flavors, making it a hearty and invigorating morning beverage.

### 5.6 Chai

A spiced tea blend originating from India. Chai combines black tea with various spices like cardamom, cinnamon, cloves, and ginger, creating a warm and aromatic flavor profile.

### 5.7 Jasmine green tea

This blend combines green tea leaves with jasmine flowers, infusing the tea with a delicate floral aroma and taste.

### 5.8 Moroccan mint

A refreshing blend, Moroccan mint combines green tea with fresh mint leaves, providing a cooling and revitalizing experience (Peterson et al., 2004).

Tea blending offers endless possibilities for creativity and innovation, allowing producers to experiment with different ingredients to develop new and exciting flavors. Additionally, blending enables tea producers to maintain consistent quality year‐round, as they can adjust the blend to accommodate variations in tea characteristics due to seasonal changes. Overall, tea blending has become an integral part of the tea industry, catering to the diverse tastes and preferences of tea drinkers worldwide and contributing to the wide array of tea choices available in the market (Spence, 2020).

## 6 HERBAL TEA

Herbal tea, or “tisanes,” refers to the infusion of plants other than *Camellia sinensis*. Although herbal tea looks like tea and is prepared similarly to tea, it is not tea at all. Tisanes or herbal teas are different from traditional teas, which are made from the *Camellia Sinensis* plant. These herbal teas are created by infusing seeds, dried leaves, nuts, grasses, barks, flowers, fruits, or other botanical ingredients to produce their distinctive flavor and health benefits. Unlike other types of tea, herbal teas do not contain caffeine and are easy to drink because of their pleasant taste. These teas can be made using a single herb or a blend of herbs that are projected to provide a specific effect such as rejuvenation, relaxation, or relief from certain conditions (Killedar & Pawar, 2017).

Tea made from herbs has been popular for a long time. Ancient Egyptian‐era documents have been found that speak of the benefits and usage of herbal tea (Kara, 2009). Before becoming popular as a daily beverage, an herbal beverage is typically appreciated as a medicinal beverage. For example, tea evolved from being a “medication” to being a “drink.” (Long et al., 2023). Not only herbal tea, but authentic tea also undergoes this influence. Around 5000–6000 years ago, in the Shen Nong dynasty of ancient China, tea leaves and other herbs were utilized for medical purposes (Pan et al., 2022). Tea drinking first appeared as a beverage in the late Zhou Dynasty (1124–222 B.C.), and it steadily gained popularity in the Qin Dynasty (221–206 B. C.) (Mujumdar, 2014).

Reports from India suggest that the leaves of five other mangrove species, including *Ceriops decandra* (Griff), *Bruguiera cylindrical* (L) Bl, Ding Hou, *Rhizopora apiculate* Blame, *R. lamarckii* Montr, and *R. mucuonata* Lam, and Blame, can be used as alternative sources of tea (Kathiresan, 1995). The leaves of various plants, including *Sorbus aucuparia*, *Epilobium angustifolium*, *Fragaria vesca*, *Filipendula ulmaria*, and *Rubus idaeus*, have been formulated to make tea by previous workers in Europe (Julkunen‐Tiitto et al., 1988). They contain several aromatic components that have medicinal properties for humans. Herbal tea might be a better description of these infusions of different plants. A variety of herbal teas are available in many Asian countries, which are made by brewing plant leaves or other parts such as flowers. One such plant is *Gymnema sylvestre* from the Asclepiadaceae family, which is commonly found in India and is used to produce a nutritious and healthy herbal tea with various medicinal properties. In general, the popularity of herbal teas is increasing nowadays (Hicks, 2001).

## 7 HEALTH BENEFITS OF HERBAL TEAS

**FIGURE 1:**
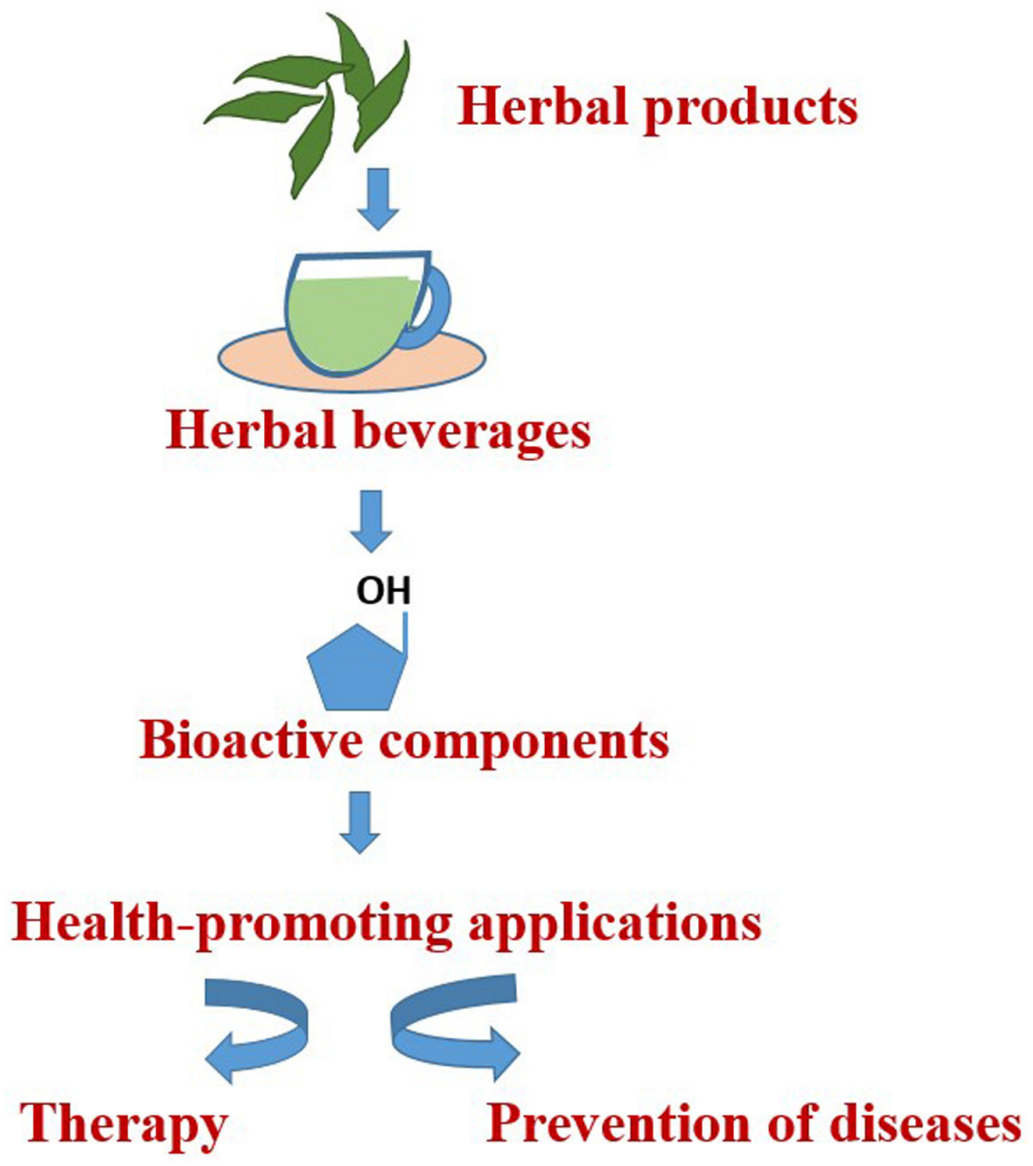
Herbal beverages promote health benefits via bioactive compounds.

Along with beverages, fruit and vegetable juices, sports drinks, and teas are considered to be functional foods (Dini, 2019). By definition, functional food lowers the chance of developing a disease or has a relevant impact on well‐being and health. The functional component of a functional food may consist of an essential macronutrient, essential micronutrient, nonessential nutrient, or nonnutritive component (Adefegha, 2018). Teas, despite having little intrinsic nutritional value, are rich in phenolic chemicals, which are associated with numerous established health benefits (Hamilton‐Miller, 1995; Marongiu et al., 2004). Various reports have documented the biological effects of polyphenols, including their anticancer, antiallergic, antibacterial, antiviral, anti‐inflammatory, estrogenic, and immune‐stimulating properties (Figure 1). They also have a reputation for having high water solubility.

Due to its growing popularity in the domestic and global markets for health advantages, herbal tea has seen an increase in consumption over the past few years. Numerous research studies have highlighted the numerous health advantages of herbal teas as well as the possibility that drinking them could lessen blood‐related irregularities (Reis et al., 2016; Sridhar & Charles, 2019). For example, red raspberry leaf tea has been traditionally employed for treating various ailments and, in specific pregnancies, to induce labor when necessary. Several researchers have explored the therapeutic advantages of herbal teas. For example, daily consumption of 1 cup (or 0.25 tsp/day for children) of chamomile herbal tea has been found to alleviate stomach discomfort and reduce anxiety. Similarly, drinking one cup of cinnamon herbal tea per day has shown the potential to reduce allergic reactions, LDL cholesterol, and blood sugar levels. For pregnant and breastfeeding individuals, it is recommended to consume 2–3 cups per day of herbal teas such as citrus peel, ginger, orange peel, lemon balm, and rosehip to maintain good health (Sridhar & Charles, 2019). According to research conducted by da Silva et al. (2017), the Brazilian berry can be utilized as a tea beverage to harness the benefits of its antioxidant content. Their findings suggest that consuming herbal teas in recommended dosages may have an impact on various diseases (da Silva et al., 2017). The following table (Table 1) presents various herbal teas, their distribution, some of their traditional uses, and the recommended daily doses associated with each type.

**TABLE 1:** Herbal teas and their traditional uses with recommended daily doses.

Common tea name	Scientific name	Used part	Traditional uses of the tea plant	Consumption doses	References
Valerian	*Valeriana officinalis* L.	Roots and rhizomes or underground stems	*Sleep aid*: It is known for its ability to promote relaxation and improve sleep quality. It is often used as a natural remedy for insomnia and other sleep disorders. *Anxiety and stress relief*: It has mild anxiolytic properties, making it helpful in reducing anxiety and stress levels. *Nervous system support*: It may help in reducing nervousness and irritability. *Menstrual discomfort*: It is used to ease menstrual cramps and discomfort associated with premenstrual syndrome (PMS). *Headache relief*: It is used to alleviate headaches, especially those caused by tension and stress. *Digestive aid*: It is used to relieve digestive issues, such as indigestion and stomach cramps.	*Infusion*: 0.5 to 1 tsp dried root in 1 cup of water. *Sleep aid*: Drink 1 cup before bed. *Anxiety*: Drink 1 cup (three times a day: TID)	Al‐Attraqchi et al. (2020), Sridhar & Charles (2019), Shinjyo et al. (2020), Dimpfel and Suter (2008), Wheatley (2005)
Peppermint	*Mentha piperita* L.	leaves	*Digestive aid*: It can help soothe indigestion, bloating, and gas, and may aid in relieving stomach cramps, discomfort, colic in infants, flatulence, diarrhea, nausea and vomiting, morning sickness, and anorexia. *Headache and migraine relief*: Peppermint tea's cooling properties may help in reducing headache and migraine symptoms. *Respiratory support*: It can act as a natural decongestant and may help ease symptoms of cold, flu, or respiratory congestion, and treat coughs and bronchitis. *Stress and anxiety relief*: The aroma of peppermint tea can have a calming effect, making it helpful in reducing stress and anxiety levels. *Muscle relaxation*: Peppermint tea may assist in relaxing muscles and can be used for relieving muscle tension and discomfort. *Oral health*: It is used to treat inflammation of oral mucosa and toothache and has antimicrobial properties that can contribute to maintaining oral health and combating bad breath. *Menstrual cramp relief*: It is used to alleviate menstrual cramps and discomfort during menstruation.	*Infusion*: 1 to 2 tsp dried leaves in 1 cup of water for 5 minutes. For irritable bowel syndrome, consider tablets (200 mg TID).	Desam et al. (2019), Fayed (2019), Kingsley (2023), Sridhar & Charles (2019), Mahendran and Rahman (2020), Shah & Mello (2004)
Nettle	*Urtica dioica L*.	Root	*Allergy relief*: It is often used as a natural remedy for seasonal allergies, such as hay fever. It may help reduce allergic reactions and alleviate symptoms like sneezing and itching. *Migraine headache relief*: Very effective in treating migraine headaches. *Anti‐inflammatory properties, joint and muscle pain*: It is used as an anodyne for arthritic conditions, muscle pain, tendonitis, nerve injuries, lack of nerve sensation, or muscle weakness. *Diuretic effects*: Its diuretic properties have been reported, which can relieve some forms of high blood pressure by reducing blood volume. *Digestive tract*: Ulceration, bleeding, diarrhea, gall bladder, and liver problems. *Blood sugar regulation*: Some studies suggest that stinging nettle tea may help regulate blood sugar levels, making it potentially beneficial for individuals with diabetes. *Iron and nutrient source*: It is rich in iron, making it a potential supplement for individuals with iron‐deficiency anemia. *Prostate health*: Stinging nettle tea has been traditionally used to support prostate health and may help alleviate symptoms of benign prostatic hyperplasia (BPH).	*Infusion*: 2.5 tsp dried root in 1 cup of water for 5–10 min. Drink 1 cup of tea BID and TID.	Farzami et al. (2003), Bhusal et al. (2022), Dhouibi et al. (2020), Sridhar & Charles (2019), Upton (2013)
Rosemary	*Rosmarinus officinalis* L.	leaves	*Digestive aid*: It is known for its digestive properties and may help with relieving the symptoms of indigestion, constipation, colitis, and stomachache. *Nervous system*: It has been associated with improved memory and mental clarity, hysteria, depression, and mental fatigue. *Circulation improvement*: It regularizes the blood pressure and retards the hardening of arteries. *Anti‐inflammatory effects*: It has anti‐inflammatory properties and may be used to relieve joint and muscle pain.	*Infusion*: 2 to 3 tsp crushed leaves in 1 cup of water for 5–10 min. Drink 1 cup of tea TID.	Ali et al. (2015), Amaral et al. (2013), Arranz et al. (2015), de Oliveira and Camargo (2019), Heinrich et al. (2006), Sridhar & Charles (2019), Peng et al. (2007)
Lemon balm	*Melissa officinalis* L.	Leaves	*Relaxation and stress relief*: It is known for its calming and soothing properties, and it is often used to promote relaxation and reduce stress and anxiety. *Sleep aid*: It is believed to have mild sedative effects, making it beneficial for individuals struggling with sleep disorders or insomnia. *Digestive aid* has been used for GIT disorders to promote digestion, and for griping pains of the belly. *Pain Relief*: It may be beneficial in reducing nerve pains and stiff necks (compress), migraine, toothaches, earaches, and headaches.	*Infusion*: 2 to 4 tsp leaf in 1 cup of water for 5–10 min. *Sleep aid*: Drink before bed. *Anxiety*: Drink 1 cup of tea BID and TID.	Cases et al. (2011), Herodež et al. (2003), Kennedy et al. (2004, 2006), Sridhar & Charles (2019)
Ginger	*Zingiber officinale*	Root	*Digestive aid*: It is used in colic and atonic dyspepsia treatment. *Nausea and morning sickness relief*: Ginger tea is often used to ease nausea and vomiting, making it a popular remedy for morning sickness during pregnancy. *Cold and Flu Relief*: It has warming properties and is used to ease cold and flu symptoms, such as sore throat, cough, and congestion. *Anti‐inflammatory effects*: It contains potent anti‐inflammatory compounds that may help reduce inflammation and alleviate joint pain, making it beneficial for individuals with arthritis or muscle soreness. *Cardiovascular health*: Heart tonic helps in preventing various heart diseases by reducing blood clotting that can lead to plaque formation or thrombosis, opening the blockage in the blood vessels, thus decreasing peripheral vascular resistance and hence blood pressure, which may help to lower high cholesterol. *Motion sickness and vertigo*: Ginger tea is believed to help alleviate motion sickness and symptoms of vertigo. *Blood Sugar Regulation*: Some research suggests that ginger may help regulate blood sugar levels, making it potentially beneficial for individuals with diabetes.	*Infusion*: 1 tsp root in 1 cup of water, take TID. *Migraine*: 1 tsp at the start of a headache, repeat in 4 h (max 4 tsp/24 h). *Childhood diarrhea*: A piece of ginger root the size of a child's little finger steeped for 5–10 min.	Akoachere et al. (2002), Alshathly (2019), Baliga et al. (2011), Dissanayake et al. (2020), Sridhar & Charles (2019), Grant and Lutz (2000), Sharma (2017)
Cinnamon	*Cinnamomum zeylanicum*	Bark	*Blood sugar regulation*: Cinnamon has been studied for its potential to help regulate blood sugar levels and improve insulin sensitivity, making it potentially beneficial for individuals with diabetes. *Anti‐inflammatory effects*: Cinnamon contains compounds with anti‐inflammatory properties that may help reduce inflammation and alleviate conditions like arthritis. *Antimicrobial properties*: Cinnamon bark has natural antimicrobial properties that can help combat certain bacteria and fungi. *Cold and flu relief*: Cinnamon bark tea has warming properties and may be used to ease symptoms of cold and flu, such as sore throat and congestion. *Cardiovascular health*: Cinnamon has been associated with potential benefits for heart health, such as reducing blood pressure and cholesterol levels.	*Infusion*: 0.5 to 3 tsp cinnamon bark in 1 cup of water for 5 min. Drink 1 cup of tea daily (may steep black teabag with bark for flavor if desired).	da Silva et al. (2017), Khan et al. (2003), Sridhar & Charles (2019), Pathak and Sharma (2021)
Fennel	*Foeniculum vulgare* Mill.	fruit	*Digestive aid*: It relieves indigestion and bloating, soothes colicky infants, and is often given to breastfeeding mothers to alleviate colic in breastfed babies and it relieves nausea and vomiting. *Respiratory support*: Fennel tea is believed to have expectorant properties and may help with respiratory issues like coughs and bronchitis. *Menstrual discomfort*: Fennel fruit tea is sometimes used to ease menstrual cramps and discomfort during menstruation. *Appetite suppressant*: Fennel tea is believed to act as an appetite suppressant and may aid in weight management. *Diuretic effects*: Fennel tea may promote urine flow and help with water retention. *Eye health*: Some cultures use fennel tea as an eye wash to soothe and cleanse the eyes, improves visual acuity, and is useful for cataract	*Infusion*: 1.5 to 4 tsp crushed fruit or seed in 1 cup of water. Take 1 cup of tea TID. *Children*: 0.04 tsp/lb/day not to exceed the adult dose.	Sridhar & Charles (2019), Mahmood et al. (2013)
Chamomile	*Chamomilla recutita* L.	Flower heads	*Relaxation and sleep aid*: It is often used to promote relaxation and improve sleep quality. *Digestive aid*: It has been used as a digestive relaxant and for various gastrointestinal disturbances including flatulence, indigestion, diarrhea, anorexia, motion sickness, nausea, and vomiting. *Sleep Aid*: It has been commonly used as a mild sedative to calm nerves and reduce anxiety to treat hysteria, nightmares, insomnia, and other sleep problems. *Skin*: It is used to treat wounds, ulcers, eczema, gout, bruises, burns, and canker sores and to soothe skin irritations, such as rashes and sunburns. *Cold and flu relief*: It has mild anti‐inflammatory properties and may help ease symptoms of colds and flu, such as sore throat and congestion has also been used to treat colic, croup, and fevers in children. *Menstrual Discomfort*: It is used to ease menstrual cramps and discomfort during menstruation. *Stress and anxiety relief*: It has a calming effect and may help reduce stress and anxiety levels. *Eye health*: It is used to treat eye infections, and disorders of the eyes including blocked tear ducts, conjunctivitis, nasal inflammation, and poison ivy.	*Infusion*: 1.5 to 5 tsp dried flower heads in 1 cup of water for 5–10 min. Drink 1 cup of tea TID. *Children*: 0.25 tsp/lb/day not to exceed the adult dose.	Peña et al. (2006), El Joumaa and Borjac (2022), Jaroenngarmsamer et al. (2021), Sridhar & Charles (2019), Motti and de Falco (2021), Sakai and Misawa (2005)
Motherwort	*Leonurus cardiaca* L.	Stems, leaves, and flowers	*Women's health*: Motherwort tea is often used to support women's health, particularly during menstruation and menopause. It may help regulate menstrual cycles, ease menstrual cramps, and alleviate menopausal symptoms such as hot flashes and mood swings. *Nervous system support*: Motherwort tea is believed to have calming properties that can help reduce anxiety, stress, and nervous tension. *Heart health*: It is used in nervous heart conditions, and as an aid in hyperthyroidism because it relieves the heart palpitations and tachycardia associated with hyperthyroidism. *Digestive aid*: Motherwort tea supports digestive health and may help alleviate indigestion and bloating. *Sleep aid*: Due to its calming effects, Motherwort tea is sometimes used as a natural remedy to improve sleep quality and aid in insomnia. *Postpartum support*: In some cultures, Motherwort tea is used to support postpartum recovery, particularly for its calming and tonic properties. *Muscle relaxation*: Motherwort tea may help relieve muscle tension and discomfort. *Anti‐inflammatory effects*: Some cultures use Motherwort tea for its anti‐inflammatory properties to help alleviate joint pain and inflammation	*Infusion*: 2 to 3 tsp dried stems, leaves, and flowers in 1 cup of water for 5–10 min. Drink 1 cup of tea TID.	Wojtyniak et al. (2013), Fierascu and Fierascu (2019), Blumenthal (2000), Huang and Xu (2021), Sridhar & Charles (2019), Patrignani et al. (2021)

## 8 THE RISING TREND OF THE HERBAL TEA MARKET

In recent years, the herbal tea market has experienced a remarkable surge in popularity, driven by evolving consumer preferences toward health and wellness (Okpiaifo et al., 2023; Puri et al., 2022). Herbal teas, also known as tisanes, have been utilized for centuries across various cultures for their medicinal properties and refreshing flavors. This section explores the current trends, growth factors, and global impact of the herbal tea market, shedding light on its burgeoning prominence in the beverage industry. The consumption of herbal tea has witnessed a significant uptick in recent times, propelled by shifting consumer attitudes toward holistic health and natural remedies. Traditionally, herbal teas were consumed primarily for their therapeutic benefits, with each herb offering unique medicinal properties. However, in contemporary times, herbal teas are increasingly valued not only for their health benefits but also for their diverse flavors and aromas (Long et al., 2024). One notable trend in herbal tea consumption is the growing popularity of home grown herbs for preparing fresh and organic herbal drinks. This trend underscores consumers' desire for authenticity, sustainability, and control over the ingredients they consume. Additionally, the rise of social media and digital platforms has facilitated the sharing of homemade herbal tea recipes, fostering a sense of community among enthusiasts. Furthermore, the COVID‐19 pandemic has accelerated the demand for herbal teas, as consumers seek immune‐boosting and stress‐relieving beverages (Wahab et al., 2023). With a growing emphasis on health and wellness, herbal teas have emerged as a preferred choice for mindful consumption, aligning with the broader trend toward conscious living.

Several factors contribute to the robust growth of the herbal tea market. Firstly, increasing awareness of the health benefits associated with herbal teas has spurred consumer interest and demand. Herbal teas are rich in antioxidants, vitamins, and minerals, offering various health‐promoting properties such as immune support, digestion aid, and stress relief (Chandrasekara & Shahidi, 2018). As consumers prioritize preventive healthcare and natural remedies, the demand for herbal teas continues to escalate. Moreover, the herbal tea market benefits from the expanding global tea industry, which is witnessing a shift toward healthier and functional beverages. With consumers seeking alternatives to sugary drinks and caffeine‐laden beverages, herbal teas offer a refreshing and nutritious option (Manteiga et al., 1997). Additionally, the rise of specialty tea shops and cafes has introduced consumers to a wide array of herbal tea blends, further fueling market growth.

The herbal tea market's impact extends beyond consumer preferences to encompass economic, environmental, and cultural dimensions on a global scale (Banerjee & Tyagi, 2024; Sousa et al., 2024). Economically, the herbal tea industry represents a lucrative market opportunity, with projected sales reaching billions of dollars in the coming years. Between 2016 and 2021, the demand for herbal tea saw a steady increase at a 5.5% value compound annual growth rate (CAGR). This growth reflects the global tea industry's rapid expansion, particularly in the herbal tea segment, driven by the growing emphasis on health and well‐being. Forecasts suggest that by 2022, the herbal tea market will reach $3.7 billion, with sales projected to grow at a CAGR of 7.1% from 2022 to 2032, reaching $7.3399 billion. By 2028, the global herbal tea market is expected to hit $4.14 billion (Insights, 2022). The report highlights the Asia Pacific region as the leading and fastest‐growing market for herbal tea during the forecast period. Countries like China and India are expected to drive significant growth in consumption, contributing substantially to the regional market. The established market for herbal tea, attributed to its recognized antiaging benefits, presents promising opportunities. Moreover, the industry is witnessing a shift toward environmentally friendly packaging, with tea bags increasingly using biodegradable or compostable materials, thus reducing carbon dioxide emissions. Additionally, the introduction of ready‐to‐drink varieties aims to further bolster sales. Innovative marketing techniques and endorsements from celebrities are anticipated to play pivotal roles in expanding the market in the years ahead (Insights, 2022).

This growth presents opportunities for tea producers, retailers, and entrepreneurs to capitalize on the rising demand for herbal teas. Furthermore, the herbal tea market plays a role in promoting environmental sustainability. Many herbal tea producers prioritize organic farming practices, sustainable sourcing, and eco‐friendly packaging, reducing their environmental footprint. Additionally, the cultivation of herbs for herbal teas contributes to biodiversity conservation and supports local farmers and communities (Chen et al., 2016; Long et al., 2023). Culturally, herbal teas hold significant importance in various societies worldwide, reflecting unique traditions, rituals, and healing practices. From traditional Chinese herbal remedies to indigenous herbal infusions in Latin America, herbal teas are deeply ingrained in cultural heritage and folklore. As the global interest in wellness and natural living continues to grow, herbal teas serve as cultural ambassadors, bridging diverse traditions and promoting cross‐cultural exchange.

In brief, the rising trend of the herbal tea market reflects a broader shift toward health‐conscious consumption and sustainable living. With consumers increasingly seeking natural remedies and wellness‐enhancing beverages, herbal teas have emerged as a favored choice worldwide. As the market continues to expand, driven by evolving consumer preferences and growing awareness of the health benefits of herbal teas, it is poised to play a significant role in the global beverage industry. By embracing authenticity, innovation, and sustainability, the herbal tea market stands to thrive, offering consumers a flavorful and nourishing alternative to traditional beverages.

## 9 CLASSIFICATION OF HERBAL TEA

Depending on the solubility of the active ingredients, herbal teas can be easily made from any part of a plant, including the flowers, roots, leaves, seeds, bark, and berries. Herbal tea can be further divided into four groups: Tea made from a single herb, such as rooibos, chamomile, or mint, is known as single herb tea. Herb mixes may include two or more herbs in addition to additional flavorings. Fruit tea, made by combining fruit bits, dried berries, herbs, and flavorings, is the most common method. Additionally, there are crossover herb mixes, which combine *Camellia sinensis* and herbs, such as black tea with rose petals, cinnamon, or sweet clove. The primary justifications for including *C. sinensis* are (i) *C. sinensis* has a strong propensity to absorb the flavors of herbs; (ii) to incorporate *C. sinensis*'s nutritional advantages, and (iii) to enhance the infusion's color (Hui, 2004). Functional herb blends are herbal concoctions made with specific goals in mind, such as weight loss, detoxification, increased energy, or general health promotion. Regular herbal tea blends can provide the advantages of functional blends. The crucial concept is that herbal tea blends or preparations depend on the cooperative action of many plant metabolites (Cseke et al., 2006). Additionally, this category of tea is typically enriched with additional elements like minerals or vitamins.

The four groups of herbal tea can also be separated. First, herbal tea has some health advantages in addition to a fantastic flavor and pleasant aroma. Generally, this tea is not harmful, thus it is acceptable for regular consumption. Ginger (*Zingiber officinale* Roscoe) is one of the Thai herbal teas in this category, Baibuabok (*Centella asiatica* (Linn.) Urban), and other teas made of flowers such as Pigul (*Mimusops elengi* Linn.) and Sarapee (*Mammea siamensis* Kosterm). Second is herbal tea like Rang‐jued (*Thunbergia laurifolia*) which has a little medicinal effect. These herbal teas have no negative side effects when consumed twice daily, once in the morning and once at night. The final category of herbal tea includes beverages meant to have a medicinal effect, such as laxatives, that is, Makhamkeak (*Senna alexandrina* P. Miller.) and Chumheadted (*Cassia alata* (L.) Roxb.). In this category of teas, consumption should be monitored because a protracted period is more detrimental than beneficial. Both of the aforementioned tea samples stimulate the gut, thus extended ingestion could interfere with or even interrupt our body's natural cycles (Saetan, 2018).

## 10 PREPARATION OF HERBAL TEA

Herbal tea can traditionally be made by using one of two methods. (i) Infusion, which is a method used to extract constituents from herbaceous tissues such as flowers, roots, leaves, or soft fruits. It involves steeping these plant parts in water at various temperatures, offering a wide range of options. These plant parts typically have a relatively high water content, ranging from 60% to 95%. Infusion is considered a mild physical condition for extraction. Moreover, (ii) decoction, which is a method that involves using more forceful physical extraction techniques, such as boiling water and longer extraction times. It is primarily used to extract active compounds from woody or highly lignified herbaceous tissues like barks, roots, twigs, and certain dry fruits. These plant parts typically have a relatively low water content, ranging from 5% to 50%. Due to its intensive extraction process, decoction is considered more effective in extracting desired constituents from such plant materials (Cseke et al., 2006). In Chinese society, this procedure typically begins with the extraction of a plant or plants using a large amount of water for several minutes or even hours, followed by the concentration of the extract. However, different cultures and personal preferences dictate how different herbal teas are made around the world. Most herbal teas are made by adding hot water and letting it soak for a few minutes before drinking. To achieve their maximum therapeutic impact, some herbal teas, like chamomile, must steep for at least 15 minutes in a closed container (Hosen, 2023). Herbal infusions can be prepared easily by steeping the herbs in hot water, and they can also be customized with the addition of milk, honey, sugar, or other sweeteners to enhance the flavor. However, the process of drinking traditional tea involves a refined art of sipping. In Chinese culture, tea is often prepared by steeping tea leaves in hot water for specific durations. For green tea, the recommended steeping time is typically between 20 and 60 seconds at temperatures ranging from 70 to 80°C. Oolong tea is steeped at temperatures between 80 and 90°C, while black tea is steeped at 100°C. The tea leaves can be steeped multiple times, with each infusion adding its unique flavor profile, before ultimately being discarded (Venditti et al., 2010). Chinese folks always discard the first infusions of oolong and green tea due to their high astringency (Horžić et al., 2009). Green tea is a favorite beverage among the Japanese, who like to steep it twice or three times in hot water. While in the West, people like to sip black tea with milk or sugar and a cup of boiling water (100°C) (Venditti et al., 2010).

## 11 CHEMICAL COMPOSITION OF HERBAL TEA

**FIGURE 2:**
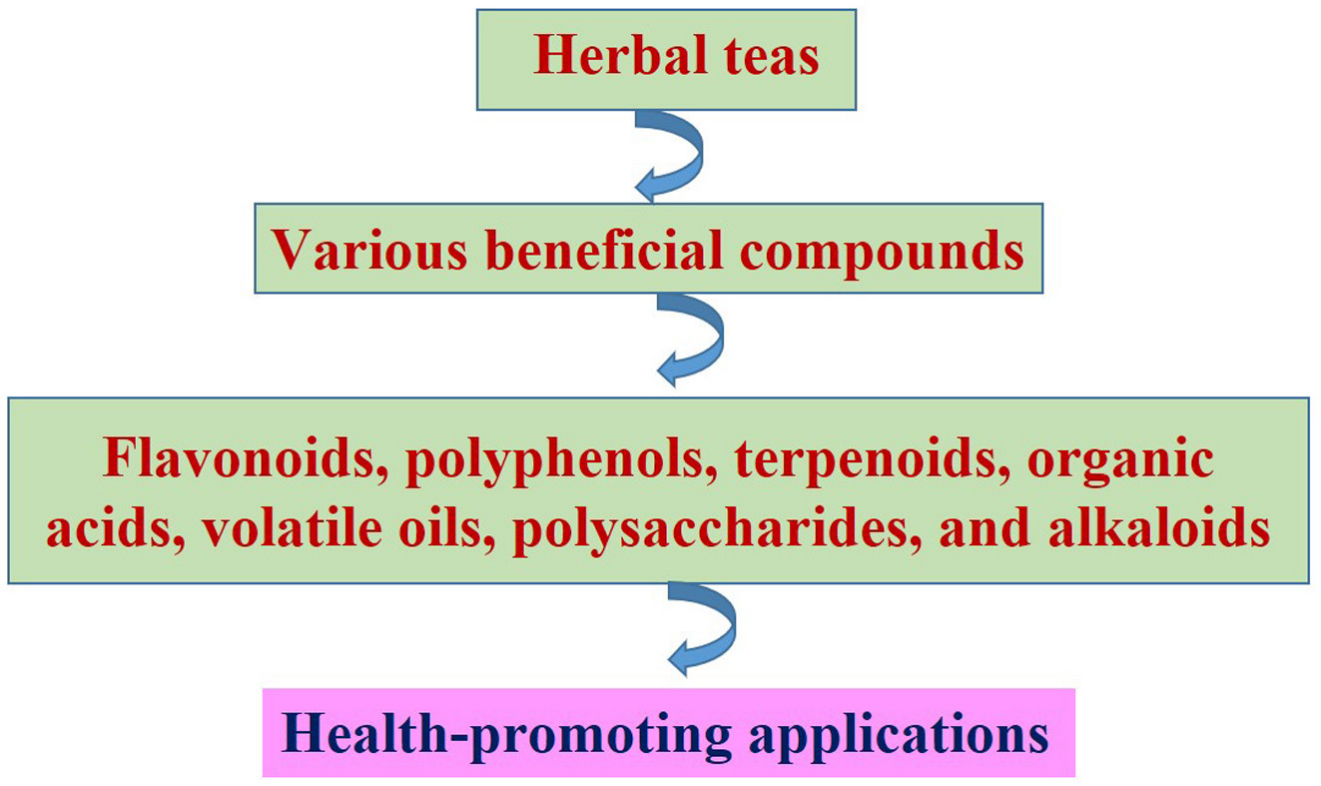
Herbal teas contain various healthy components.

Herbal teas are known to contain various beneficial compounds (Figure 2), including flavonoids, polyphenols, terpenoids, organic acids, volatile oils, polysaccharides, and alkaloids (Ye et al., 2015). However, research is scarce regarding the specific chemical constituents and their effects on herbal teas. To provide a comprehensive overview, we have compiled a table (Table 2) summarizing the commonly reported natural active ingredients found in different herbal teas:

**TABLE 2:** Chemical composition of herbal teas.

Compound category	Herbal tea example	Active components	Reference
Flavonoids	Rooibos, Kudingcha, Hong Dou Shan ( *Taxus chinensis* ) leaf tea, *Combretum micranthum* G. Don tea, *Isodon amethystoides* (Benth.) Hara tea, mountain tea, plateau tea, and She Medicine Fresh herb tea	Luteolin, rutin, kaempferol, quercetin, myricetin, dihydromyricetin, and apigenin	Arries et al. (2017), Bai et al. (2010), Chen et al. (2016), Cooper et al. (2005), Duan et al. (2021), Gao et al. (2020), Gong et al. (2020), Guzelmeric et al. (2020), Kpemissi et al. (2020)
Polyphenols	Arabian jasmine, balsam pear, barley grass, guava, Hardy rubber tree, Japanese persimmon, and jobs tears	Catechin, gallic acid, (−)gallocatechin, sinapinic acid, caffeic acid, (−)epicatechin, gallocatechin, chlorogenic acid, ellagic acid, and corilagin	Chen et al. (2020), Gao et al. (2020), Liang et al. (2007), Ning and Han (2013), Nurfaradilla & Saputri (2020), Toda (2011)
Terpenoids	Chamomile tea, *Rubus corchorifoliu* L. f.	Oleanolic acid, ursolic acid, friedelin, akebia saponin D, and ilexgenin B	Chen et al. (2017), Orimadegun et al. (2018), Raal et al. (2012)
Volatile Oils	Kainari tea, vine tea, rooibos, kuding tea, yerba mate tea, hawk tea	Eugenol, cinnamic aldehyde, myristicin, cinnamyl alcohol, (−)perilla aldehyde, and linalool	Bampali et al. (2018)
Alkaloids	Jasmine‐scented tea, barley tea, Yunnan Kucha tea, Gouqiye tea	Caffeine, hordenine, theacrine, glycine betaine, atropinol, and scopolamine	Dang et al. (2011), Liang et al. (2007), Lin et al. (2020), Yang et al. (2007)
Other compounds (*L*‐rhamnose monohydrate, DL‐arabinose, *L*‐glutamine, tannins, steroids, anthraquinone, polysaccharides, proteins, amino acids, and vitamins) have been isolated. Vitamin C, vitamin B1, vitamin B2	*Strobilanthes crispus* leaf tea, *Siraitia grosvenorii*	Tannins, steroids, anthraquinone, polysaccharides, proteins, amino acids, vitamins (vitamin C, vitamin B1, and vitamin B2), L‐rhamnose monohydrate, DL‐arabinose, and L‐glutamine	Dujnič et al. (2021), Ismail et al. (2000)

## 12 TEA MANUFACTURING AND PREPARATION

To make teas like peppermint, chamomile, and rose tea, the herb is simply dried until the moisture level is lower than 10%. To remove all the contaminants and debris, the freshly collected leaves will go through washing and cleaning. The leaves will be dried indoors at room temperature to reduce their moisture content after cleaning. Heater air at a temperature of 40°C might help speed up the withering process (Mujumdar, 2014). Tea's aroma is produced through the withering process, which also makes the leaves more malleable for the subsequent step (Hara, 2001). The withering leaves of the *Camellia sinensis* plant are fed into a rolling machine where they are twisted. To start the fermentation process, the cell membranes of the leaves are broken and battered. The tea leaves can either be dried immediately after rolling to generate partially fermented tea (oolong tea), or they can be let to continue fermenting to produce fermented tea (black tea). For herbal teas, cutting is typically used to reduce the size of the leaves and increase their surface area, which encourages the fermentation process. The herbal tea leaves are often fermented before being dried to produce the final product. The polyphenol oxidase‐induced oxidative chemical reaction on the polyphenols in tea leaves is the most significant response in the fermentation of tea. As a result, tea with varying degrees of fermentation develops various colors, smells, and flavors (Hui, 2004). Another crucial step in the production of tea is drying, which serves as a preservation strategy for the beverage, stopping microbial development and enzymatic destruction. It is crucial to choose the right drying temperature. While too low a temperature can hasten decomposition by encouraging enzyme activity within the plant itself or even microbial attack, especially for items containing sugar, too high a temperature can result in the loss of active components. Therefore, to stop the enzymatic fermentation reaction and encapsulate the flavors inside the leaves, tea leaves are typically dried at an oven temperature of 90–95°C, with higher temperatures (>95°C) occasionally being used (Mujumdar, 2014). Tea is made according to a short process. The plant material, which is often kept in a bag, is taken from the container after steeping in hot water (between 70 and 100°C) for a short period (generally 1 to 5 minutes). The period of steeping and the degree of water temperature has an impact on the tea's “strength.” Tea can be sipped hot, warm, or cooled. Before drinking, milk and/or a sweetener like sugar or honey may occasionally be added (Hakim et al., 2000).

## 13 BENEFITS OF DRINKING HERBAL TEA

Since water is the most vital component of life, we should drink eight glasses of it every day. In the normal course of things, it may be challenging for some people to get the recommended daily intake of plain water, especially for kids. In this situation, herbal teas can be a lovely beverage and a fantastic technique to help our bodies absorb water. The amount of caffeine in tea, however, is one of the main worrying factors. Tea has a high caffeine content that can reach 23.79 g/kg (Turkmen & Velioglu, 2007).

To reduce the amount of caffeine in numerous sources, including tea and coffee, decaffeination has gained popularity. Due to the adverse effects of caffeine, which include nauseousness, anxiety, jitteriness, nervousness, a rise in blood pressure, a drop in heart rate, and an increased risk of cardiovascular disease, it is recommended that beverages contain a minimum level of caffeine content (Temple, 2009); a dose of more than 10 g (or 170 mg/kg body weight) could even be fatal (Sinija & Mishra, 2009). Since most herbal teas are known to be caffeine free in this context, this could be an alternative to tea. Additionally, herbal teas with low tannin content and no caffeine, such as rooibos and honeybush tea, are excellent for midnight beverages and anxious people (Marnewick, 2009).

The high oxalate content of tea is another health concern associated with its consumption. Someone who regularly drinks six cups of tea a day can expect to ingest anywhere from 26.46 to 98.58 mg of soluble oxalate from loose black tea, and between 17.88 and 93.66 mg from black tea bags. However, herbal teas only provide a maximum of 18.0 mg/day of soluble oxalate (Charrier et al., 2002). The oxalate level of herbal teas was also discovered to be 7 to 32 times lower than that of black tea. Consuming high levels of oxalate in the diet is believed to be a risk factor for developing calcium oxalate kidney stones, a condition known as hyperoxaluria. As a result, herbal teas are considered a better choice for individuals who have had stones, as they have a significantly lower oxalate content than black teas (McKay & Blumberg, 2006; Noonan & Savage, 1999).

The main advantage of drinking herbal teas is that they provide an alternative option to regular tea and offer unique flavors, tastes, and nutritional benefits that traditional tea may lack. For instance, chamomile tea, a well‐known herbal infusion made from flowers, contains bisabolol and chamazulene. These compounds have been found to possess anti‐inflammatory, antioxidant, and leukotriene B4 synthesis‐inhibiting properties. Peppermint tea, known for its strong aroma, has been shown to have antibacterial and antiviral properties, as well as high antioxidant and antitumor properties. It is rich in rosmarinic acid and various flavonoids, particularly eriocitrin, luteolin, and hesperidin (McKay & Blumberg, 2006).

## 14 FUTURE DYNAMICS OF HERBAL BEVERAGES

Natural products, mainly medicinal plants and their phytoconstituents, have been used to cure diseases from ancient times. Chinese people had become aware, as early as 4000–5000 years ago, that tea could cure and prevent some human diseases. Today hundreds of millions of people around the world drink tea. Green tea from the medicinal plant has many health benefits. The use of fresh and dried medicinal plants to prepare tea that is popularly consumed for its pleasant taste and, most notably, its perceived health benefits dates back to antiquity. However, in recent years, consumers have become more health conscious regarding the prevention and treatment of disease, which has led to the development of plant‐based health products, including herbal teas. Therefore, developing herbal beverage products from medicinal plants with various medicinal properties is an essential and timely action for the future development of herbal beverages. Different previous research indicates that medicinal plants were good anticancer, radioprotective, genoprotective, hepatoprotective, antimicrobial, antiaging, and antioxidant agents besides being nontoxic toward humans and animals, which provides ample justifications for the requisite development of herbal beverage products. Besides, medicinal plants represent an inexpensive but high‐quality therapeutic plant source of polyphenols and antioxidants with suitable biological activities, an essential attribute of green tea. More recently, various studies reported that green tea with high‐quality polyphenols could reduce cancer risk. Several studies initiated by many other laboratories have suggested that polyphenols found in medicinal plants may reduce the risk of various diseases, including cancers, in humans. Furthermore, multiple reports have shown an inverse association of tea consumption with cancer development. Moreover, effectively exploiting a high commercial potential medicinal plant as an herbal beverage requires carefully designed consumer profiling, focusing on the health benefits of the new herbal beverage. Interestingly, medicinal plants possess such vital multihealth‐related functions, and some of these have already been proved at the preclinical level in animal models, indicating the opportunities for developing commercially viable herbal drinks from medicinal plants. These unique features of herbal beverages will make the product competitive in the market, especially among patients who undergo treatment. Despite the apparent health benefits of medicinal plants and traditional usage being widely reported, there are relatively no commercial brands of this plant material available, and this plant material still needs to be utilized to develop new herbal beverages.

## 15 CONCLUSION

In conclusion, this review article has offered an engaging exploration of the fascinating world of tea and herbal beverages. This review article has provided valuable insights into these beverages' diverse and cherished realm by delving into their origins, trade history, and health benefits. Moreover, we have shed light on the chemical composition of herbal teas, highlighting their rich content of bioactive compounds. By comprehending teas' market dynamics, classification, preparation, and manufacturing processes, we deepen our appreciation for these drinks that hold cultural significance and offer potential health‐promoting properties. This comprehensive review serves as a valuable resource, celebrating the enduring allurement of tea and herbal beverages in human lives and cultures. As we continue to unravel the scientific discoveries and traditional wisdom surrounding these beverages, we gain a deeper understanding of their profound impact on human well‐being and enjoyment.

## AUTHOR CONTRIBUTIONS

**Marcin Bryła:** Writing – review and editing (equal). **Marek Roszko:** Writing – review and editing (equal). **Sreenivasan Sasidharan:** Conceptualization (equal); supervision (equal); visualization (equal); writing – review and editing (equal). **Syed Amir Ashraf:** Formal analysis (equal); software (equal); validation (equal); writing – review and editing (equal). **Hisham‐Sultan‐Alkatib Huda:** Conceptualization (equal); writing – original draft (equal); writing – review and editing (equal). **Nazia Binti Abdul Majid:** Validation (equal); writing – original draft (equal); writing – review and editing (equal). **Mohd Adnan:** Software (equal); validation (equal); visualization (equal); writing – review and editing (equal). **Yeng Chen:** Validation (equal); writing – original draft (equal); writing – review and editing (equal). **Marek Kieliszek:** Writing – review and editing (equal).

## CONFLICT OF INTEREST STATEMENT

The authors declare that they have no known competing financial interests or personal relationships that could have appeared to influence the work reported in this paper.

## Data Availability

No data were used for the research described in the article.
